# The Effect of Oral Consumption of Probiotics in Prevention of Heart Injury in a Rat Myocardial Infarction Model: a Histopathological, Hemodynamic and Biochemical Evaluation

**DOI:** 10.18869/acadpub.ibj.21.3.174

**Published:** 2017-05

**Authors:** Jafar Sadeghzadeh, Abedin Vakili, Hamid Reza Sameni, Mahdi Shadnoush, Ahmad-Reza Bandegi, Mahdi Zahedi Khorasani

**Affiliations:** 1 Students’ Research Center, School of Medicine, Semnan University of Medical Sciences, Semnan, Iran; 2 Research Center and Department of Physiology, Faculty of Medicine, Semnan University of Medical Sciences, Semnan, Iran; 3 Research Center of Nervous System Stem Cells, Department of Anatomy, Faculty of Medicine, Semnan University of Medical Sciences, Semnan, Iran; 4 Department of Clinical Nutrition Faculty of Nutrition and Food Technology, Shahid Beheshti University of Medical Sciences, Tehran, Iran; 5 Semnan University of Medical Sciences, Faculty of Medicine, Semnan, Iran; 6 Department of Biochemistry, Faculty of Medicine, Semnan University of Medical Sciences, Semnan, Iran

**Keywords:** Myocardial injury, Tumor necrosis factor-alpha, Probiotics, Isoproterenol, Oxidative stress

## Abstract

**Background:**

Despite the emerging evidence on beneficial effects of probiotics on the cardiovascular system, their impact on the management of ischemic heart diseases and the possible mechanism(s) have not been elucidated.

**Methods:**

Four viable probiotics bacterial strains, including *Bifidobacterium breve*, *Lactobacillus casei*, *Lactobacillus bulgaricus*, and *Lactobacillus acidophilus*, at the concentrations of 2×10^6^ colony-forming units/ml, were orally administered to the rats daily for 14 days before the induction of infarct-like myocardial injury using isoproterenol. Subsequently, 24 h after myocardial injury, the right carotid artery and the left ventricle were catheterized for recording blood pressure and cardiac parameters. At the end of the experiment, the heart was removed for the evaluation of histopathological and biochemical parameters, as well as tumor necrosis factor-alpha (TNF-α) assay.

**Results:**

The induction of acute myocardial injury resulted in significant (*P*≤0.01) left ventricular (LV) dysfunction, as shown by an increase in LV end-diastolic pressure and a decrease in LV dp/dt max, LV dp/dt min, LV systolic pressure, and blood pressure, as compared with normal rats. Pretreatment with viable probiotics significantly reduced lipid peroxidation and TNF-α level and improved cardiac function (*P*<0.01).

**Conclusion:**

This study shows that viable probiotics have a cardioprotective effect on infarct-like myocardial injury through suppressing TNF-α and oxidative stress damage in a rat model. Probiotic supplements may be used as a new option for prophylaxis in patients at the risk of ischemic heart disease in future.

## INTRODUCTION

At present, many populations in the world are at moderate or high risk of cardiovascular disease, which has become one of the primary causes of mortality and morbidity[1]. Probiotics are beneficial microorganisms that may promote and protect health through various mechanisms. Several studies have demonstrated the positive effects of probiotics in the prevention and/or management of certain diseases, including diarrhea and irritable bowel syndrome[2], liver dysfunction[3], hyper-cholesterolemia[4], as well as colon cancer, peptic ulcer, Crohn’s disease, and lactose intolerance[5]. In addition, there is growing evidence suggesting that probiotics are useful for the cardiovascular system. Zhao *et al*.[6] have reported that the administration of a protein (p75) purified from *Lactobacillus rhamnosus* GG, 30 min before the induction of ischemia, significantly attenuates myocardial ischemic injury in rats. Similarly, Lam *et al*.[7] have demonstrated the cardioprotective effects of probiotics against myocardial infarction in rats. Similarly, Gan *et al*.[8] have reported that a six-week oral administration of the probiotic *L. rhamnosus* GR-1 following coronary occlusion can attenuate the post-infarction myocardial hypertrophy and heart failure in rats. In addition, another animal study has shown that spontaneous treatment hypertensive rats with probiotic-fermented purple sweet potato yogurt is helpful[9]. Recently, Costanza *et al*.[10] have shown that short-term probiotic therapy in human patients with chronic systolic heart failure results in improvement of left ventricular (LV) ejection fraction as well as reduction in left atrial diameter.

Although recent evidence has indicated that probiotics can potentially be helpful in cardiac failure and ischemic heart diseases, these studies are still limited, and the efficacy of probiotics in the prevention of cardiovascular diseases such as myocardial ischemia has not been assessed yet. In this study, we have investigated the effect of probiotics and their possible mechanism of action in the prevention or attenuation of cardiac damage in an isoproterenol (ISO)-induced infarct-like myocardial injury in rats.

## MATERIALS AND METHODS

### Animals

A total of 32 male Wistar rats (250±50 g) obtained from the Breeding Colony of Semnan University of Medical Sciences (Semnan, Iran) were used in this study. The animals were kept in a standard cage and had free access to food and water. All experiments were performed in accordance with the Research Ethics Committee of Semnan University of Medical Sciences (SUMS) with ethical code number: IR.SEMUMS.REC.1394.98 and with the national guidelines for conducting animal studies. Sodium thiopental was provided by Kwality Pharmaceuticals Pvt. Ltd., India.

### Experimental design and protocol

All 32 animals were randomly divided into five groups. The first group (n=8) included the rats that received saline as sham treatment (normal control group). The second group was normal rats (n=4) that were given probiotics at a concentration of 2×10^6^ colony-forming units (CFUs)/mL. The third group of rats (n=9) received 85 mg/kg ISO (ISO group). The fourth group (n=5) was pretreated with inactivated probiotics by gastric gavage daily for 14 days, then received ISO (ISO+inactivated probiotics group [(ISO+In.P)]). Rats in the fifth group (n=6) were pretreated with probiotics at the concentration of 2×10^6^ CFUs/mL by gastric gavage daily for 14 days and then received ISO (ISO+probiotics [ISO+P]). After 24 h of the second dose of ISO injection and the induction of acute infarct-like myocardial injury, all rats were anesthetized using sodium thiopental (80 mg/kg intraperitoneally), and the femoral artery and left ventricle of the heart were cannulated for recording blood pressure, hemodynamic parameters, and cardiac function. Two tissue samples of tissue from the apex of the left ventricle were removed immediately for the measurement of biochemical and histological parameters. A known weight of the ventricular tissue was homogenized in 5.0 mL of 0.1 M Tris-HCl buffer (pH 7.4) solution. The homogenate was centrifuged, and the supernatant was used for the estimation of various biochemical parameters such as tumor necrosis factor-alpha (TNF-α), malondialdehyde (MDA) content, and thiobarbituric acid reactive substances.

### Probiotic strains and preparation

In this study, we used a combination of four viable probiotic bacteria strains, such as *Bifidobacterium breve*, *Lactobacillus casei*, *Lactobacillus bulgaricus* (*Lactobacillus delbrueckii subsp. bulgaricus*), and *Lactobacillus acidophilus* with 10^9^ CFU/g colony count, which was prepared in the laboratory scale at Neurophysiology Research Center of Shahid Behest University of Medical Sciences, Tehran, Iran. The 2×10^6^ CFU/mL[11] dosage was prepared by dissolving 500 mg probiotics in 250 mL saline. From these prepared solutions, 1 mL was administered via gastric gavage daily for 14 days. For the inactivated probiotics, probiotic bacteria were killed in an oven at a temperature of 80°C for 15 min.

### Induction of myocardial injury

Acute myocardial injury, an infarct-like myocardial lesion, was induced with the subcutaneous injection of ISO (85 mg/kg) at 24-h intervals for two consecutive days.

### Histopathological measurement

At the end of the experiment, under deep anesthesia, tissue from the LV cardiac apex was removed and fixed in 10% buffered formalin solution. Heart tissue sections (5 μm thickness) were cut and stained using hematoxylin-eosin for histopathological examination under a light microscope (200×). The slides were evaluated for necrosis, inflammatory cell infiltration, and edema. A minimum of 10 fields for each slide were examined and graded for the severity of changes as follows: Grade 1 (−): absence of inflammation, edema and necrosis; Grade 2 (+): focal areas of inflammation, edema, and necrosis; Grade 3 (++): patchy areas of inflammation, edema, and necrosis; Grade 4 (+++): confluent areas of inflammation, edema, and necrosis; and Grade 5 (++++): massive areas of inflammation, edema, and necrosis[12]. The examiner was unaware of the animal experimental groups.

### Biochemical parameter and tumor necrosis factor-alpha assay

The supernatants of heart tissue were used for biochemical analyses. The total protein content in the heart tissue homogenate was determined using the Bradford’s method[13]. MDA content (as a lipid peroxidation index) and thiobarbituric acid reactive substances were measured as described previously[14]. The colorimetric ferric reducing/antioxidant power (FRAP) assay was used to measure the total antioxidant capacity[15]. The levels of TNF-α were measured by an ELISA method using a Rat TNF-α ELISA Kit (Biorbyt, United Kingdom).

### Measurement of hemodynamic and cardiac parameters

Twenty-four hours after the second ISO injection and under thiopental sodium anesthesia, a polyethylene cannula (PE-50) was inserted into the right common carotid artery for recording the heart rate and arterial blood pressure (Powerlab System; AD Instruments, Australia). Then the polyethylene cannula was gently advanced into the LV lumen for the measurement of LV systolic pressure (LVSP), LV end-diastolic pressure (LVEDP), the maximum rate of LV pressure increase (LV dp/dt max; contraction velocity), and the maximum rate of LV pressure decline (LV dp/dt min; relaxation velocity). These parameters were continuously monitored and recorded using a Power Lab data acquisition system (AD Instruments, Australia).

### Statistical analysis

For the comparison of the groups regarding LVSP, LVEDP, LV dp/dt min, LV dp/dt max, TNF-α, and oxidative stress biomarkers, one-way ANOVA and Dunnett’s post-hoc test were used. Results were presented as mean±SEM. Differences were considered significant at *P*<0.05 (SigmaStat 2.0; Jandel Scientific, Erkrath, Germany).

## RESULTS

### Effect of probiotic treatment on LV function and hemodynamic parameters

**Fig. 1:**
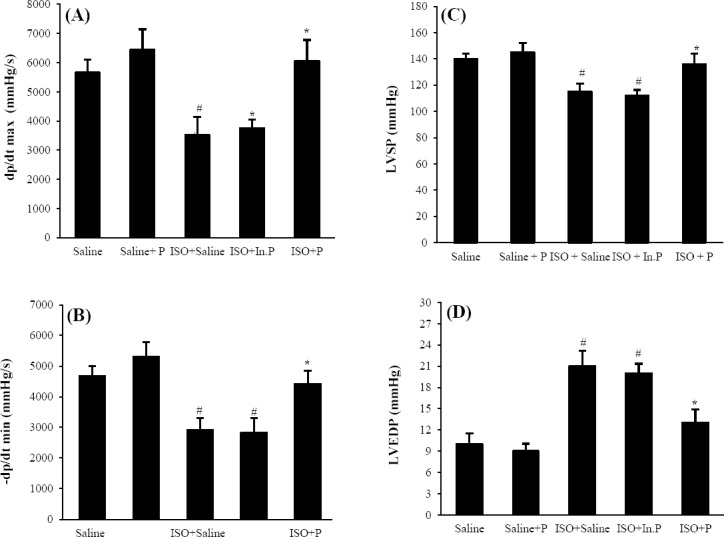
Left ventricular cardiac function indices. (A) Maximum rate of left ventricular (LV) pressure increase (+LV dp/dt max; contraction velocity); (B) maximum rate of LV pressure decline (-LV dP/dt min; relaxation velocity); (C) left ventricular systolic pressure (LVSP); (D) left ventricular end-diastolic pressure in Saline, Saline+P, ISO+Saline, ISO+In.P, and ISO+P. Values are mean±SEM. ^#^*P*<0.001 from respective saline and saline+P groups; ^*^P<0.001 as compared with ISO+Saline and ISO+In.P groups. In.P, inactivated probiotics, P, probiotics; ISO, isoproterenol

**Fig. 2:**
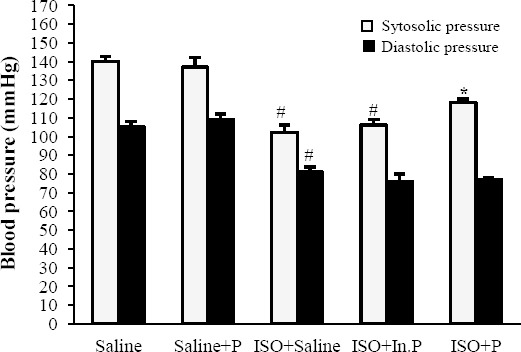
Systolic and diastolic pressure in rats pretreated with Saline, Saline+P, ISO+Saline, ISO+In.P, and ISO+P. Values are mean±SEM. ^*^P<0.01 as compared with ISO+saline and ISO+In.P groups. ^#^*P*<0.001 from respective saline and saline+P groups. In.P, inactivated probiotics; P, probiotics; ISO, isoproterenol

**Fig. 3:**
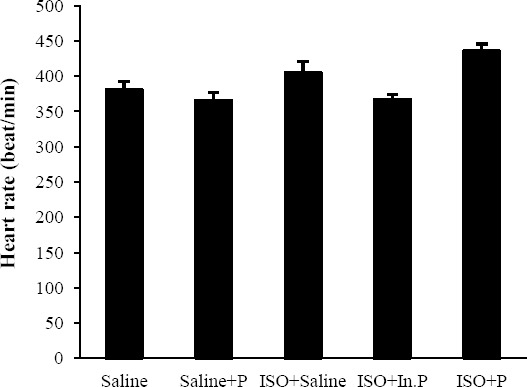
Heart rate in rats pretreated with Saline, Saline+P, ISO+Saline, ISO+In.P, and ISO+P. Values are mean±SEM. In.P, inactivated probiotics; P, probiotics; ISO, isoproterenol

After 24 h of ISO-induced myocardial injury, a significant increase in LVEDP and decrease in LV dp/dt max, LV dp/dt min, LVSP, and blood pressure (*P*≤0.01) was seen in ISO+saline group compared to control groups (Figs. 1 and 2). The oral administration of probiotic bacteria significantly (*P*≤0.007) improved all these parameters compared with the ISO+saline group. Heart rate was not significantly changed (*P*>0.05) in the ISO+saline group in comparison to the other groups (Fig. 3). The raise in LVEDP in rats treated with ISO+saline was significantly (*P*<0.05) attenuated by pretreatment with the probiotics bacteria (Fig. 1D). There was no significant difference between ISO+saline and ISO+In.P in LV function, as well as histopathological and hemodynamic parameters (*P*>0.05).

### Effect of probiotic pretreatment on histo-pathological scores

**Fig. 4:**
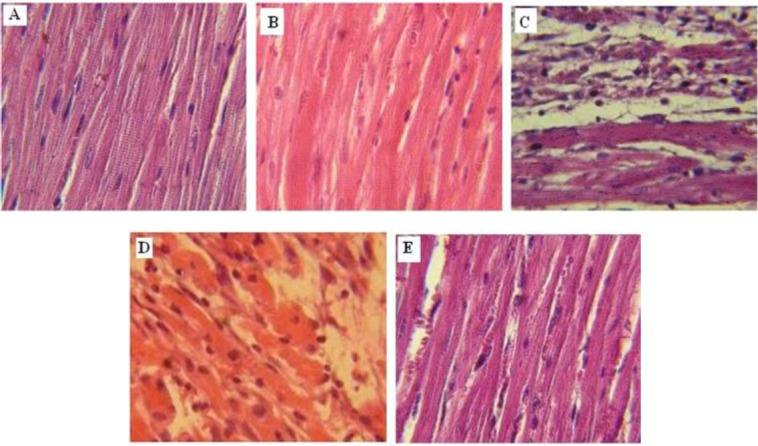
Photomicrograph showing the histopathological changes in heart tissue in rats pretreated with (A) Saline, (B) Saline+P, (C) ISO+saline, (D) ISO+In.P, and (E) ISO+P. H&E (400× magnification). In.P, inactivated probiotics; P, probiotics

The result of histopathological examinations for the normal (saline) and probiotic-treated normal (saline+P) groups are shown in Table 1. In the normal rats, histological sections of heart tissues indicated regularly arranged myocardial fibers with clear striations and without myocardial necrosis and edema (Fig. 4). ISO treatment (ISO+saline) induced widespread subendocardial necrosis with edema, leukocyte infiltration, and the splitting of cardiac myofibrils compared with the normal group (Table 1 and Fig. 4). Pretreatment with probiotics (ISO+P) considerably reduced the ISO-induced myocardial necrosis, edema, and infiltration of inflammatory cells (Table 1, Fig. 4).

**Table 1:** Effect of pretreatment with probiotics on histopathological alteration in myocardial injury induced by isoproterenol in rats

Groups	Myonecrosis	Inflammation	Edema
Saline	-	-	-
Saline+P	-	-	-
ISO+saline	+++	+++	++++
ISO+In.P	+++	+++	++++
ISO+P	+	+	++

(-), the absence of inflammation, edema, and myonecrosis; (+), the focal area of inflammation, edema, and myonecrosis; (++), patchy areas of inflammation, edema, and myonecrosis; (+++), confluent areas of inflammation, edema, and myonecrosis; (++++), massive areas of inflammation, edema, and myonecrosis. In.P, inactivated probiotics. P, probiotics; ISO, isoproterenol

### Effect of probiotic pretreatment on lipid peroxidation

**Fig. 5:**
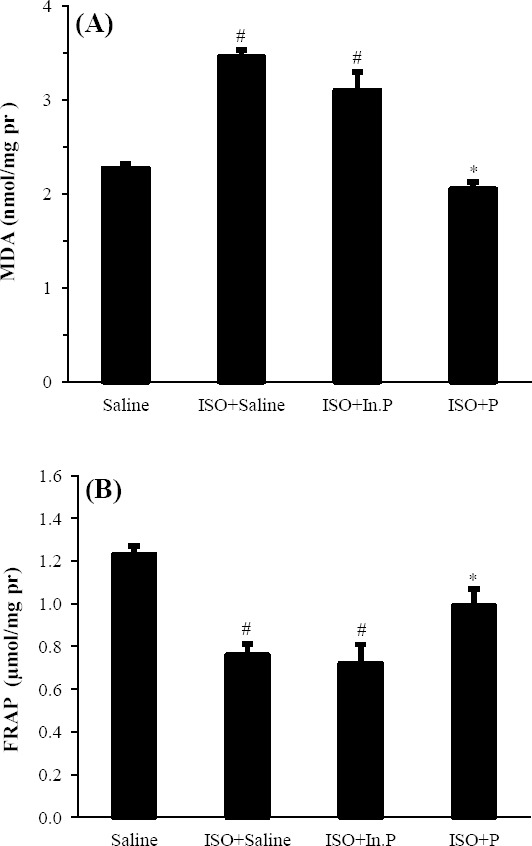
Oxidative stress biomarker including (A) malondialdehyde (MDA) and (B) ferric reducing/antioxidant power (FRAP) levels in rats pretreated with Saline, Saline+P, ISO+ saline, ISO+In.P, and ISO+P. ^#^*P*<0.01 from respective saline value; *P<0.01 as compared with ISO+saline, and ISO+In.P groups. Values are mean±SEM. In.P, inactivated probiotics; P, probiotics; ISO, isoproterenol

After 24 h of myocardial injury, the MDA levels were significantly increased, and the total antioxidant capacity reduced in the heart tissue only in ISO-treated (ISO+saline) and ISO+In.P rats compared with the normal control (saline). Treatment with probiotics significantly (*P*<0.01) decreased the MDA content, as a lipid peroxidation marker, and increased the total antioxidant capacity or FRAP index in the heart tissue (Fig. 5).

### Effect of probiotic pretreatment on pro-inflammatory cytokine; TNF-α

**Fig. 6:**
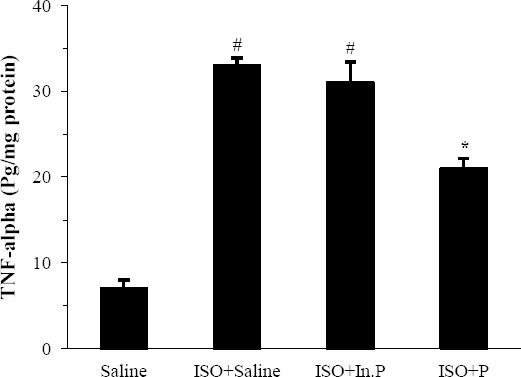
TNF-α levels in rats pretreated with Saline, ISO+saline, ISO+In.P, and ISO+P. Values are mean±SEM. ^#^*P*<0.01 from respective saline value; ^*^P<0.01 as compared with ISO+saline and ISO+In.P groups. In.P, inactivated probiotics; P, probiotics; ISO, isoproterenol

TNF-α concentrations were significantly increased in the ISO+saline and ISO+In.P-treated groups as compared with the saline group (*P*<0.001, Fig. 6). Treatment with the probiotic significantly reduced the levels of TNF-α in heart tissue (*P*<0.001, Fig. 6).

## DISCUSSION

There is little information about the host-microbiome interaction and its influence on the health of the cardiovascular system in humans. For the first time, our study showed that a two-week pretreatment with the combination of four probiotic strains of bacterial groups, such as *B. breve*, *L. casei*, *L. acidophilus*, *L. rhamnosus*, and *L. bulgaricus*, in a rat model of ISO- induced myocardial injury, reduced heart damage and improved cardiac function (LVEDP, LVSP, LV dp/dt max, LV dp/dt min). The hemodynamic and histopathological parameters were also improved significantly.

A widely used model to study the possible protective effects of agents on myocardial infarction is coronary ligation, in which the heart is exposed to a left thoracotomy incision. This model is more complex and has a higher incidence of morbidity and mortality unrelated to myocardial infarction, and a mortality rate of approximately 50%[16]. Therefore, in the present study, we have used a reliable and effective model to generate a myocardial injury similar to that of myocardial infarction[17]. Using ISO, the infarct-like myocardial lesion along with the cardiac dysfunction, and other toxic symptoms mimicking acute myocardial infarction in human was induced in rats[16,17].

ISO-induced myocardial injury is mainly attributed to the production of free radicals, which cause cardiac dysfunction, increased lipid peroxidation, and depletion of endogenous antioxidants[12]. Lipid peroxidation is one of the common basic deteriorative reactions following myocardial injury. MDA has been already identified as a major lipid peroxidation product and is a sensitive biomarker of oxidative stress[18]. Our results showed that pretreatment with probiotic bacteria reduced lipid peroxidation and increased the total antioxidant capacity in the heart tissue. Consistent with these results, Amaretti *et al*.[19] have recently reported that probiotic bacteria demonstrate antioxidant activity *in vitro* and *in vivo*. In addition, they showed that the administration of probiotic bacteria was able to protect rats against doxorubicin-induced oxidative damage in a dose-dependent manner, which further confirms the findings of the present study[19]. Our results also support the hypothesis that probiotic bacteria may enhance and augment cellular antioxidant defenses in the host. Thus, at least a part of the cardioprotective effects of probiotics may be related to the enhancement of antioxidant enzyme synthesis or activity in cardiac tissue. This supposition is supported by previous studies in rat models that showed probiotics supplemented with *L. casei*, *L. acidophilus*, and *Bifidobacterium lactis* enhance the synthesis and augmentation of enzymes with antioxidant activity via alteration in gene expression levels[20,21].

Recent investigations have indicated that probiotics may inhibit pro-inflammatory cytokines such as TNF-α, stimulate the production of anti-inflammatory cytokine interleukin-10, and activate the anti-apoptotic Akt pathway[22,23]. In the gut epithelium, probiotics interact with toll-like receptors via activated transcription factors that can regulate inflammatory responses[24,25].

It has been reported that the activation of pro-inflammatory cytokines such as TNF-α has an important role in worsening ischemic injury following myocardial infarction[26,27]. Our study showed that probiotic pretreatment noticeably reduced TNF-α concentration in the heart tissue, which suggests that a part of the cardioprotective effect of probiotics observed in the present study might be related to their anti-inflammatory properties and the attenuation of TNF-α synthesis. However, more investigation is needed to explore other mechanisms that might be responsible for the protective effects of probiotics against myocardial injury. The current study also showed that myocardial infarction results in various histopathological changes, including muscle fibers necrosis along with inflammatory cell infiltration, edema, and the fragmentation of muscle fibers. Pretreatment with probiotic bacteria considerably improved these histopathological changes, which include the relative preservation of myocardial fiber structure and morphology. This finding confirms the cardioprotective effect of probiotics against myocardial ischemia injury in rats.

In addition, in the current study, the effect of inactivated probiotics (heat-killed bacteria) on ISO-induced myocardial injury was investigated. Our findings indicated that there is no difference between ISO-treated (ISO+saline) and ISO+In.P in cardiac function, histological assay, and hemodynamic parameters. This interesting result showed that the cardioprotective effects observed in this study are related to viable probiotics. These findings reveled that the viability of probiotics is probably needed for preclinical or clinical benefits. However, this hypothesis requires further exploration. Our data are consistent with other experimental research, showing that viable probiotics are more effective than non-viable probiotics[28,29]. Although some preclinical studies demonstrate that viability is not necessary for all probiotic effects[30,31], there are very few data to confirm the clinical efficacy of non-viable probiotics. The current study also showed that pretreatment with probiotic bacteria noticeably improved both LV systolic and diastolic dysfunction and hemodynamic parameters. In line with our findings, Lam *et al*.[7] reported that there is a relationship between changes in the intestinal microbiota and the severity of myocardial infarction in rats. In addition, studies have shown that probiotic therapy in rats[8] and humans[9] attenuated myocardial hypertrophy and heart failure and improved LV ejection fraction, which further confirms the results of the present study.

In summary, the current study showed that probiotics have a cardioprotective effect against heart ischemic injury through the attenuation of TNF-α and oxidative stress in a rat model of infarct-like myocardial injury. We suggest that the viable probiotic supplements may be useful in the prevention or management of cardiac injury in patients at risk of myocardial infarction. However, more preclinical and clinical research studies are needed to elucidate the role of probiotics as a new option in the prophylaxis and the management of cardiovascular disease.
